# Chinese visceral adiposity index predicts all-cause mortality in patients with type 2 diabetes, heart failure, and chronic kidney disease: a retrospective cohort study

**DOI:** 10.1186/s13104-025-07531-6

**Published:** 2025-10-29

**Authors:** Xiang Zhao, Dan Zhang, Bing Zhu, Yuanhao Yao, Lingxiao Geng, Xiang Ma, Zhenyan Fu, Yitong Ma

**Affiliations:** 1 Second Department of Coronary Heart Disease, The First Affiliated Hospital of Xinjiang Medical University, Urumqi, Xinjiang China; 2 The Second Affiliated Hospital of Xinjiang Medical University, Urumqi, Xinjiang China; 3 Department of Coronary Heart Disease, The First Affiliated Hospital of Xinjiang Medical University, Urumqi, Xinjiang China

**Keywords:** Chinese visceral adiposity index, Visceral adiposity, Heart failure, Type 2 diabetes mellitus, Chronic kidney disease

## Abstract

**Objective:**

This study aimed to evaluate the prognostic value of the Chinese Visceral Adiposity Index (CVAI) for all-cause mortality in the high-risk population with coexisting type 2 diabetes mellitus (T2DM), heart failure (HF), and chronic kidney disease (CKD).

**Results:**

Over a median 26-month follow-up among 442 patients, 194 deaths occurred. The highest CVAI tertile (T3) exhibited a significantly elevated mortality risk (adjusted hazard ratio [HR] = 2.806, 95% confidence interval [CI] 1.848–4.261, *P* < 0.001), demonstrating a linear dose-response relationship. CVAI provided significant incremental predictive value beyond left ventricular ejection fraction (LVEF) and N-terminal pro-B-type natriuretic peptide (NT-proBNP). Subgroup analysis revealed a significant association in patients with LVEF < 40% but not in those with LVEF ≥ 40% (P for interaction = 0.049).

**Supplementary Information:**

The online version contains supplementary material available at 10.1186/s13104-025-07531-6.

## Background

Heart failure (HF) is a multisystem syndrome affecting cardiac, endocrine, musculoskeletal, renal, and vascular systems. Despite therapeutic advances, HF remains linked to high mortality/morbidity, poor life quality, and functional decline [1]. HF patients often exhibit comorbidities like type 2 diabetes mellitus (T2DM), chronic kidney disease (CKD), and chronic obstructive pulmonary disease, contributing to multimorbidity and worse outcomes [2]. Metabolic comorbidities critically influence HF prognosis. Metabolic remodeling—a key HF pathophysiological feature—involves impaired energy metabolism, reducing high-energy phosphate production in cardiac/skeletal muscle and exacerbating HF progression [3]. This remodeling encompasses altered substrate utilization, tricarboxylic acid cycle defects, and oxidative phosphorylation deficits [4], synergizing with chronic inflammation to worsen HF and comorbidities.

First, T2DM increases HF risk 2- to 4-fold [5], and HF cohorts show diabetes prevalences up to 50% [6], reflecting bidirectional disease interplay. Second, CKD and HF are closely interrelated. CKD markedly elevates cardiovascular risk, especially in diabetes [7, 8]. Diabetic hyperglycemia promotes CKD progression via glomerular hypertension and podocyte apoptosis. Concurrent CKD and HF substantially increase mortality [9]. Epidemiologically, HF, T2DM, and CKD form a complex “cardio-renal-metabolic syndrome.” This triad is not merely additive: T2DM-driven advanced glycation end products (AGEs) accelerate myocardial fibrosis through receptor for AGEs (RAGE)-mediated oxidative stress, mitochondrial dysfunction, and apoptosis [10, 11]. CKD activates the renin-angiotensin-aldosterone system (RAAS), inducing ventricular remodeling [12], while HF-induced hypoperfusion accelerates diabetic nephropathy—creating a vicious cycle that elevates mortality beyond individual diseases. In HF, T2DM and CKD exhibit bidirectional amplification [13]. CKD onset temporally associates with HF events [9], and HF increases subsequent renal risk (and vice versa), particularly in placebo-treated patients [14], highlighting bidirectional disease burden.

 Visceral Adipose Tissue (VAT) acts as a pathogenic hub in metabolic comorbidities. VAT expansion increases proinflammatory cytokines (e.g., interleukin-6 [IL-6], tumor necrosis factor-alpha [TNF-α]), driving insulin resistance [15, 16], and reduces adiponectin, exacerbating metabolic dysregulation [17]. The Chinese Visceral Adiposity Index (CVAI) was selected as the primary adiposity metric in this study for several reasons. First, as a indices developed and validated specifically in Chinese cohorts [18], it is optimized to capture visceral adipose dysfunction and its associated metabolic risks in this population, potentially offering superior performance over indices derived from Western populations (e.g., Visceral Adiposity Index, VAI). Second, compared to the Lipid Accumulation Product (LAP), which incorporates only waist circumference and triglycerides, CVAI integrates additional clinically relevant parameters—namely age, body mass index (BMI), and high-density lipoprotein cholesterol (HDL-C). This comprehensive profile provides a more nuanced reflection of metabolic health and age-related risk. Finally, we prioritized CVAI over simpler anthropometric measures like the waist-to-hip ratio (WHR) because WHR measurements can be unreliable in patients with heart failure and chronic kidney disease, where fluid overload and edema may significantly distort hip and waist measurements, leading to misclassification. CVAI, a noninvasive VAT surrogate, predicts diabetic kidney disease (DKD) development [19] and albuminuria progression [20]. CVAI also outperforms other obesity indices in cardiovascular disease (CVD) risk assessment [21, 22]. However, existing studies focus on single diseases, neglecting comorbidity interactions. CVAI’s value in HF, particularly the triple-comorbidity population, remains unexplored. This study evaluates CVAI’s prognostic utility for all-cause mortality in T2DM-HF-CKD patients.

## Methods

**Fig. 1:**
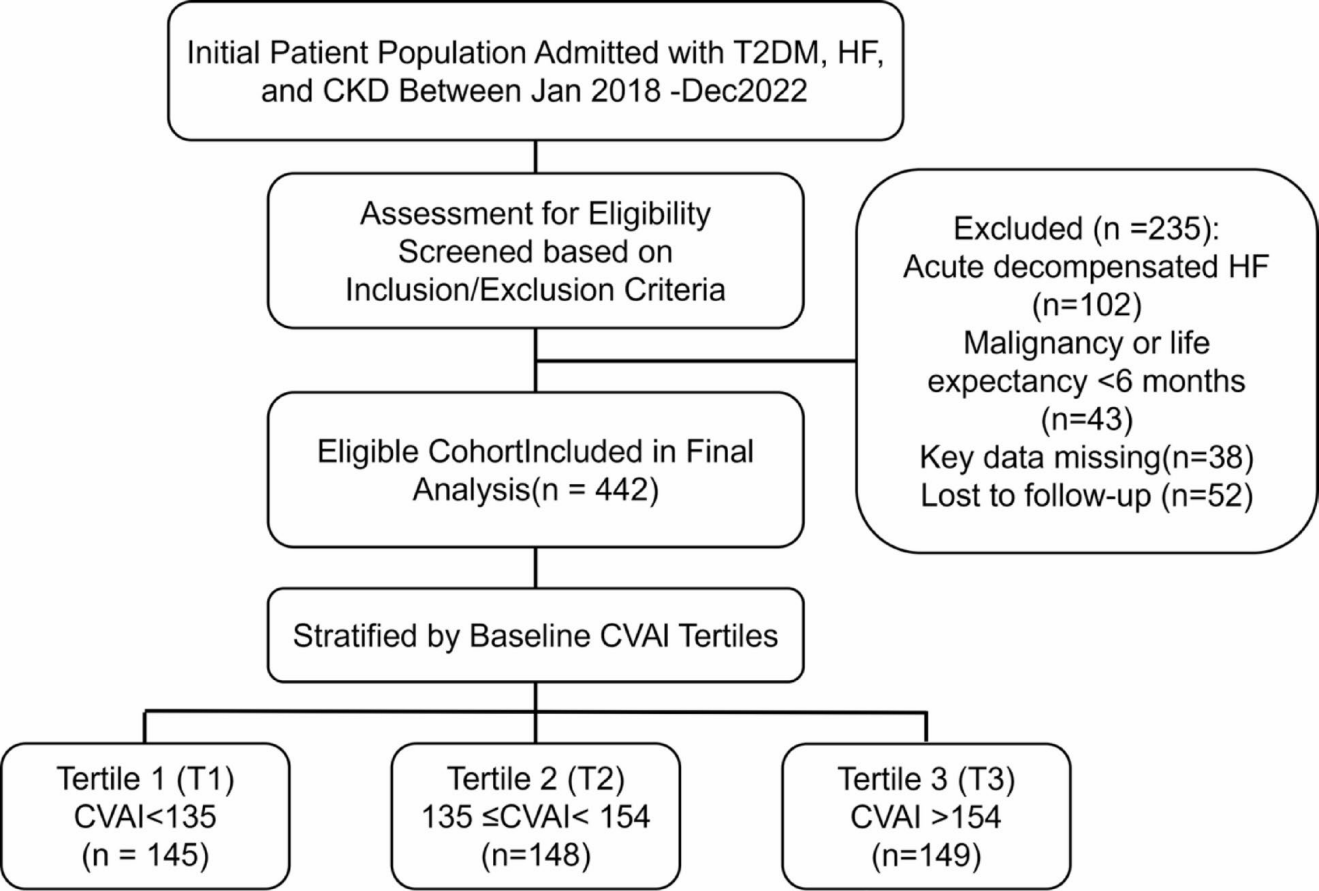
Flowchart of patient selection for the retrospective cohort study. T2DM, type 2 diabetes mellitus; HF, heart failure; CKD, chronic kidney disease; CVAI, Chinese Visceral Adiposity Index

Study Population: Consecutive adults (≥ 18 years) hospitalized with T2DM [23], chronic HF [24], and CKD (eGFR < 60 mL/min/1.73 m² >3 months) [25] were enrolled (2018–2022). Exclusions: acute HF decompensation, active malignancy/life expectancy < 6 months, or incomplete data. Ethics approval (No. 240104-02) waived informed consent for anonymized retrospective data (Fig. 1).

### Data Collection

Demographics, clinical parameters (including NYHA class), laboratory results (lipid profiles, HbA1c, NT-proBNP, eGFR), and echocardiographic LVEF were systematically documented. Body mass index (BMI) and CVAI [26] (sex-specific formula) were calculated.

### Endpoint

The primary endpoint was all-cause mortality. Cardiovascular mortality, a secondary endpoint, was defined as death attributable to acute myocardial infarction, sudden cardiac death, heart failure, stroke, or other direct cardiovascular causes. Deaths were verified via medical records and telephone follow-up until December 31, 2023.

### Statistical analysis

Baseline CVAI tertiles defined groups (T1: <135; T2: 135–154; T3: ≥154). Continuous variables summarized as median (IQR) or mean ± SD; categorical as n (%). Inter-group comparisons used ANOVA/rank-sum or chi-square tests. Survival differences assessed via Kaplan-Meier/log-rank. Multivariable Cox models (adjusted hierarchically) estimated mortality risk, Model 1: Adjusted for sex and age. Model 2: Model 1 + smoking, hypertension, prior myocardial infarction. Model 3: Model 2 + eGFR, LDL-C, LVEF, NT-proBNP, HbA1c, NYHA class. Additionally, to account for competing risks and evaluate cause-specific mortality, a Fine-Gray subdistribution proportional hazards model was employed to assess the association between CVAI tertiles and the risk of cardiovascular death, with non-cardiovascular death treated as a competing event. The potential nonlinear relationship between CVAI as a continuous variable and all-cause mortality was examined using restricted cubic splines (RCS) with 4 knots placed at the 5th, 35th, 65th, and 95th percentiles. The linearity assumption was tested by comparing the model fit with and without the nonlinear components using a likelihood ratio test. Predictive performance evaluated by ROC analysis. To assess the incremental predictive value of CVAI beyond traditional risk factors, we calculated the Net Reclassification Improvement (NRI) and Integrated Discrimination Improvement (IDI). Prespecified subgroups included sex, age, NYHA class, and LVEF. Sensitivity analyses excluded eGFR < 15 mL/min/1.73 m² or early deaths (< 6 months).

## Results

This retrospective cohort study included 442 patients with comorbid T2DM, HF, and CKD, comprising 313 males (70.8%) with a mean age of 65 ± 9 years. Participants were stratified into three groups by baseline CVAI tertiles: T1: 145 patients (CVAI < 135), T2: 148 patients (135 ≤ CVAI < 154), T3: 149 patients (CVAI ≥ 154). As shown in Table 1, significant differences existed across groups in sex distribution, smoking prevalence, BMI, WC, TG, and HDL-C (all *P* < 0.05). Other baseline characteristics were comparable (Table 1).

**Table 1:** Comparison of baseline characteristics by CVAI tertile groups

	Tertile 1 (*n* = 145)	Tertile 2 (*n* = 148)	Tertile 3 (*n* = 149)	*P*
CVAI	120.96 (106.50, 128.27)	143.82 (138.28, 149.89)	163.85 (158.90, 169.60)	< 0.001
Male	63 (43.4)	120 (81.1)	130 (87.2)	< 0.001
Age	65 ± 9	65 ± 9	67 ± 9	0.247
Smoking	13 (9.0)	22 (14.9)	42 (28.2)	< 0.001
Hypertension	129 (89.0)	126 (85.1)	135 (90.6)	0.325
Previous MI	45 (31.0)	50 (33.8)	47 (31.5)	0.865
BMI, kg/m2	23.3 (21.4, 25.0)	25.3 (23.9, 27.07)	28.1 (26.1, 31.6)	< 0.001
WC, cm	88 (82, 91)	95 (91, 97)	98 (97, 101)	< 0.001
eGFR, ml/min/1.73 m²	42 (29, 50)	39 (26, 47)	37 (28, 51)	0.537
SBP, mmHg	127 ± 23	131 ± 28	124 ± 21	0.807
DBP, mmHg	75 ± 13	79 ± 13	73 ± 16	0.864
FBG, mmol/L	9.3 (6.6, 12.9)	9.3 (6.0, 13.2)	9.4 (6.8, 14.4)	0.583
TG, mmol/L	1.06 (0.78, 1.60)	1.31 (0.92, 1.87)	1.38 (1.04, 1.94)	< 0.001
TC, mmol/L	3.24 (2.65, 3.95)	3.32 (2.76, 4.19)	3.38 (2.77, 4.10)	0.423
HDL-C, mmol/L	0.97 (0.73, 1.16)	0.90 (0.71, 1.07)	0.84 (0.67, 0.99)	0.001
LDL-C, mmol/L	1.98 (1.52, 2.48)	2.11 (1.66, 2.87)	2.17 (1.62, 2.66)	0.145
LVEF, %	38 ± 8	38 ± 8	38 ± 6	0.190
NT-proBNP, pg/ml	3500 (1820, 8600)	4174 (1848, 9995)	3876 (1737, 9580)	0.554
HbA1c, %	7.7 (6.6, 9.4)	7.9 (6.9, 9.3)	7.9 (7.0, 9.4)	0.556
NYHA class				
II	26 (17.9)	15 (10.1)	41 (28.3)	0.319
III	78 (53.8)	88 (59.5)	45 (30.4)	
IV	41 (28.3)	90 (60.4)	42 (28.2)	

Abbreviations: CVAI, Chinese Visceral Adiposity Index; MI, Myocardial Infarction; BMI, Body Mass Index; WC, Waist Circumference; eGFR, Estimated Glomerular Filtration Rate; SBP, Systolic Blood Pressure; DBP, Diastolic Blood Pressure; FBG, Fasting Blood Glucose; TG, Triglycerides; TC, Total Cholesterol; HDL-C, High-Density Lipoprotein Cholesterol; LDL-C, Low-Density Lipoprotein Cholesterol; LVEF, Left Ventricular Ejection Fraction; NT-ProBNP, N-terminal pro-B-type Natriuretic Peptide; HbA1c, Glycated Hemoglobin; NYHA class, New York Heart Association Functional Class

**Fig. 2:**
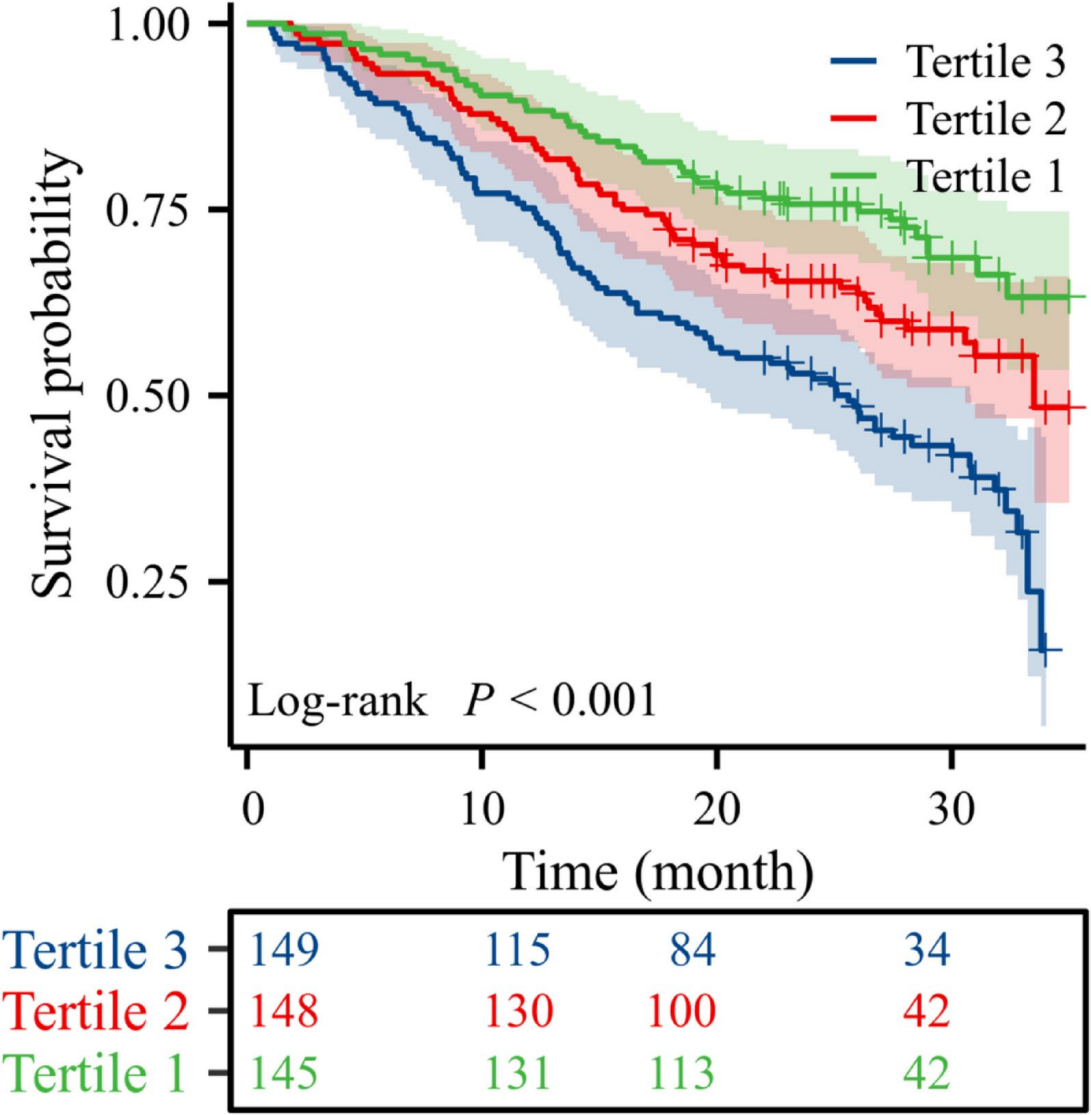
Kaplan-Meier Survival Curves by CVAI Tertile Groups

**Fig. 3:**
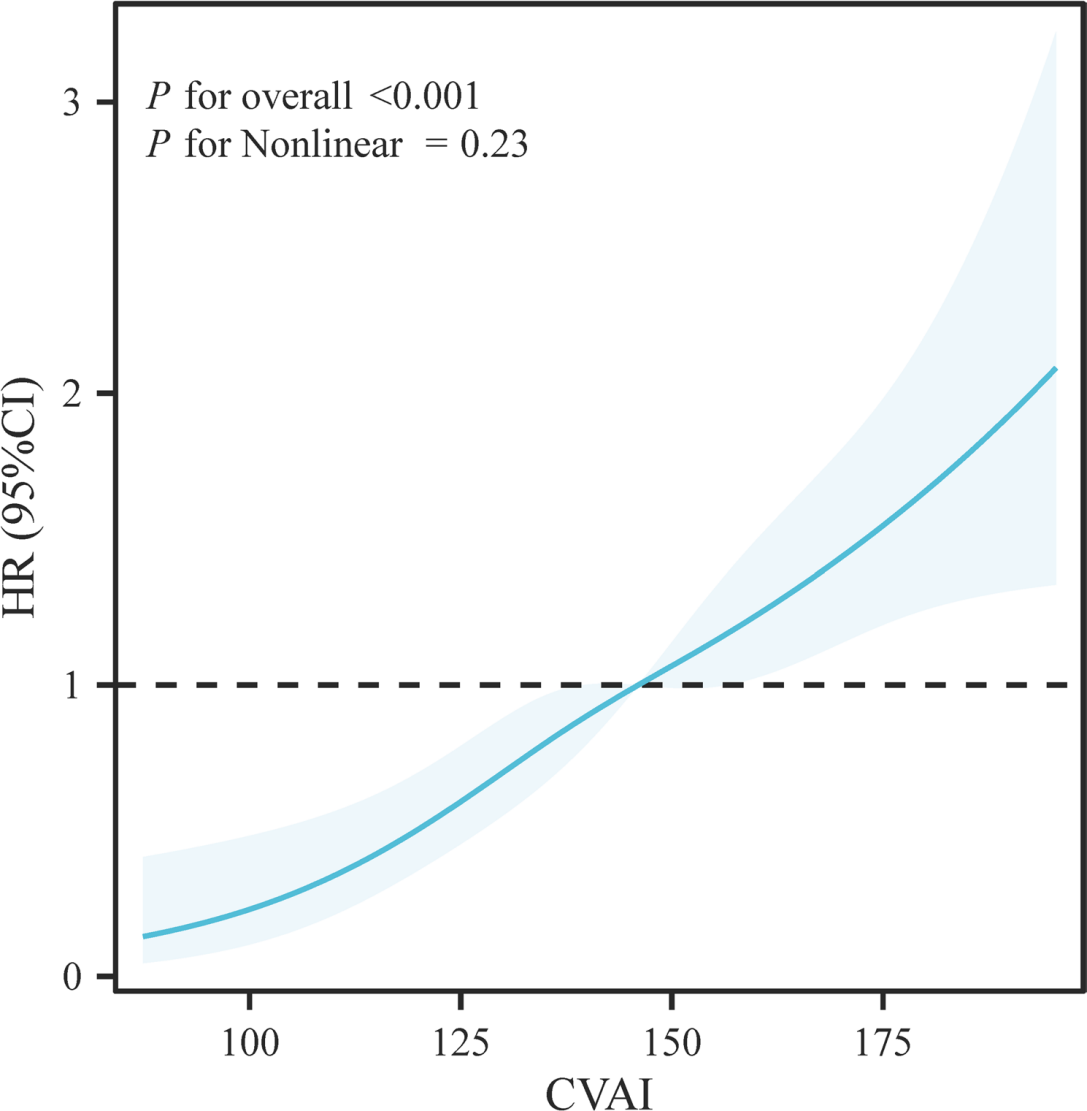
Restricted Cubic Spline Plot of the Association Between CVAI and All-Cause Mortality Dose-response relationship between CVAI and the risk of all-cause mortality. The solid line represents the HR, and the shaded area represents the 95% confidence interval. The model was adjusted for age, sex, smoking, hypertension, prior myocardial infarction, eGFR, LDL-C, LVEF, NT-proBNP, HbA1c, and NYHA class

Over a median follow-up of 26 months (IQR: 15–30 months), 194 all-cause deaths occurred. Kaplan-Meier analysis revealed significantly different cumulative survival rates (log-rank *P* < 0.001), with the T3 (high-CVAI) group exhibiting the poorest survival (Fig.2). Cox regression demonstrated increased mortality risk in higher CVAI groups (reference: T1; unadjusted HR = 2.514, 95% CI 1.748–3.617, *P* < 0.001). Based on clinical practice, we adjusted for potential confounders through sequential models. Results confirmed that higher CVAI levels remained independently associated with increased mortality risk after adjustment (adjusted HR = 2.806, 95% CI 1.848–4.261, *P* < 0.001; Table 2). To address the potential influence of non-cardiovascular mortality, a competing risk analysis was performed. The Fine-Gray model demonstrated that high CVAI (Tertile 3) remained significantly associated with an increased risk of cardiovascular-specific mortality (subdistribution hazard ratio [sHR] = 2.59, 95% CI 1.60–4.21, *P* < 0.001), with results highly consistent with the primary Cox analysis for all-cause mortality (Supplementary Table 1). Furthermore, when modeled as a continuous variable using restricted cubic splines, CVAI demonstrated a significant, approximately linear, positive association with the risk of all-cause mortality (P for overall association < 0.001; P for nonlinearity = 0.228). This dose-response relationship, visualized in Fig.3 , indicates a monotonically increasing risk of death with higher CVAI levels.

**Table 2:** Cox regression analysis of CVAI tertiles and all-cause mortality

	Tertile 1	Tertile 2 (HR, 95%CI)	Tertile 3 (HR, 95%CI)	*P* for trend
Unadjusted	Ref.	1.491 (1.009–2.203)	2.514 (1.748–3.617)	< 0.001
Model 1	Ref.	1.536 (1.012–2.331)	2.645 (1.768–3.956)	< 0.001
Model 2	Ref.	1.559 (1.025–2.371)	2.690 (1.794–4.035)	< 0.001
Model 3	Ref.	1.611 (1.050–2.472)	2.806 (1.848–4.261)	< 0.001

Model 1: Adjusted for sex and age. Model 2: Model 1 + smoking, hypertension, prior myocardial infarction. Model 3: Model 2 + eGFR, LDL-C, LVEF, NT-proBNP, HbA1c, NYHA class. HR, Hazard Ratio; Confidence interval

ROC analysis indicated that CVAI had the largest area under the curve (AUC) for mortality prediction (AUC = 0.657, 95% CI 0.607–0.708) compared to age, BMI, WC, TG, and HDL-C (Table 3). The incremental predictive value analysis (Supplementary Table 2) demonstrated that adding NT-proBNP to the baseline LVEF model yielded an NRI of 0.154 (95% CI 0.068–0.259), indicating significant improvement in risk classification. Further incorporation of CVAI increased the NRI to 0.321 (95% CI 0.062–0.413), demonstrating significant incremental predictive value. The Integrated Discrimination Improvement index similarly supported this finding, with the addition of CVAI resulting in an IDI of 0.123 (95% CI 0.013–0.259). These results indicate that CVAI provides important incremental prognostic value beyond traditional heart failure biomarkers.

**Table 3:** ROC analysis for predicting all-cause mortality by CVAI and conventional parameters

	AUC (95%CI)	*P*
Age	0.487 (0.433–0.542)	0.648
BMI	0.627 (0.575–0.679)	< 0.001
WC	0.627 (0.575–0.679)	< 0.001
TG	0.544 (0.490–0.598)	0.112
HDL-C	0.587 (0.533–0.641)	0.002
CVAI	0.657 (0.607–0.708)	< 0.001

Abbreviations: BMI, Body Mass Index; WC, Waist Circumference; TG, Triglycerides; HDL-C, High-Density Lipoprotein Cholesterol, CVAI, Chinese Visceral Adiposity Index; AUC, area under the curve

Subgroup analyses (Table 4) showed significant associations between CVAI and mortality risk in males, females, patients aged < 65/≥65 years, NYHA class II–III, NYHA class IV, and LVEF < 40% (all *P* < 0.05). No significant association was observed in patients with LVEF ≥ 40% (HR = 1.006, 95% CI 0.993–1.019, *P* = 0.388), with a significant interaction effect (P for interaction = 0.049). Sensitivity analyses after excluding patients with eGFR < 15 mL/min/1.73 m² (*n* = 30) yielded consistent results (HR = 1.018, 95% CI 1.011–1.024). Further exclusion of early events (< 6-month follow-up; *n* = 32) also showed consistent associations (HR = 1.017, 95% CI 1.011–1.024), with no significant link in the LVEF ≥ 40% subgroup (HR = 1.008, 95% CI 0.995–1.021, *P* = 0.237) (Table 5).

**Table 4:** Subgroup analysis of association between CVAI and all-cause mortality

	HR (95%CI)	*P*	*P* for interaction
Sex			0.304
Male	1.017 (1.009–1.026)	< 0.001	
Female	1.030 (1.018–1.042)	< 0.001	
Age			0.330
Age ≥ 65	1.023 (1.014–1.031)	< 0.001	
Age < 65	1.019 (1.007–1.031)	0.001	
LVEF			0.049
LVEF < 40% (*n* = 274)	1.019 (1.012–1.027)	< 0.001	
LVEF ≥ 40% (*n* = 168)	1.006 (0.993–1.019)	0.388	
NYHA			0.721
NYHA II-III (*n* = 299)	1.016 (1.008–1.024)	< 0.001	
NYHA IV (*n* = 143)	1.036 (1.021–1.051)	< 0.001	

HR represents the hazard ratio per 1-standard deviation (SD) increase in CVAI. All models were adjusted for age, sex, smoking status, hypertension, prior myocardial infarction, eGFR, LDL-C, NT-proBNP, HbA1c, and NYHA class. P for interaction was derived from Wald test of the interaction term. HR, Hazard Ratio; Confidence interval

**Table 5:** Sensitivity analysis of CVAI and a ll-cause mortality risk

	HR (95%CI)	*P*
Primary analysis (*n* = 442)	1.019 (1.013–1.025)	< 0.001
EGFR < 15 mL/min/1.73 m² was excluded (*n* = 30)		
Overall cohort (*n* = 412)	1.018 (1.011–1.024)	< 0.001
LVEF < 40% (*n* = 257)	1.018 (1.011–1.025)	< 0.001
LVEF ≥ 40% (*n* = 155)	1.006 (0.993–1.018)	0.368
Early deaths (< 6 months) was excluded (*n* = 32)		
Overall cohort (*n* = 410)	1.017 (1.011–1.024)	< 0.001
LVEF < 40% (*n* = 247)	1.018 (1.010–1.026)	< 0.001
LVEF ≥ 40% (*n* = 163)	1.008 (0.995–1.021)	0.237

Abbreviations: EGFR: Estimated Glomerular Filtration Rate; HR, Hazard Ratio; Confidence interval

## Discussion

This study pioneers the prognostic validation of the CVAI in patients with the triple comorbidity of T2DM, HF, and CKD. Our results demonstrate that patients with elevated CVAI levels exhibit the lowest cumulative survival rates. Critically, CVAI retains independent predictive value for all-cause mortality even after adjusting for key clinical indicators. The AUC for CVAI exceeds that of any individual component in its calculation formula, highlighting its clinical utility as an integrative metabolic biomarker.

Our study establishes CVAI as a significant independent risk marker for mortality in T2DM-HF-CKD patients. It identifies a high-risk phenotype characterized by visceral adiposity dysfunction. While this association does not prove causality, it suggests that targeting visceral obesity could be a therapeutic strategy. Consequently, patients with elevated CVAI may be ideal candidates for intensified multimodal management, including lifestyle interventions and pharmacotherapies with proven efficacy in reducing visceral adiposity and improving cardiorenal outcomes, such as SGLT2 inhibitors and GLP-1 receptor agonists. Future interventional studies are needed to determine if specifically targeting a reduction in CVAI translates into a survival benefit, which would solidify its role as a modifiable treatment target.

Obesity, particularly pathological VAT accumulation, constitutes a key pathological basis for cardio-renal-metabolic disease progression. VAT expansion is closely associated with chronic low-grade inflammation, recognized as a core pathological mechanism underlying metabolic syndrome and cardiovascular disease [27]. Studies indicate that chronic inflammation within VAT promotes insulin resistance and other metabolic abnormalities through the secretion of multiple proinflammatory cytokines and adipokines [27]. VAT accumulation independently predicts coronary artery calcification progression [28], suggesting VAT directly influences cardiovascular disease advancement through associated metabolic risk factors. Furthermore, VAT accumulation correlates with left ventricular dysfunction [29], further supporting its pathological role in cardiovascular disease. VAT deposition is also intimately linked to multiple components of metabolic syndrome, including insulin resistance, hyperglycemia, hypertriglyceridemia, and low HDL-C levels [30].

The T2DM-HF-CKD cohort examined in this study represents the advanced stage of cardio-renal-metabolic syndrome, where VAT accelerates mortality risk through multiple mechanisms. The significantly increased all-cause mortality risk observed in the high-CVAI group (reflecting VAT dysfunction) aligns closely with these established pathways. As an optimized VAT indicator for Chinese populations, CVAI integrates age, WC, BMI, TG, and HDL-C. This integration explains why CVAI’s predictive efficacy significantly outperforms traditional single metrics, more effectively capturing the dysfunctional state of VAT and providing a more comprehensive quantification of metabolic risk. Research validating CVAI as an optimized VAT indicator for Chinese populations demonstrates its superiority in detecting VAT dysfunction [31]. Wu et al. repeatedly measured CVAI in participants without baseline CVD during their study period. Follow-up results revealed that CVAI levels were closely associated with the risk of new-onset HF in this population [31]. Moreover, CVAI demonstrates predictive potential for other metabolism-related diseases. Studies indicate CVAI correlates with CKD risk in Korean populations, outperforming other indicators regardless of sex [32]. In a study of 8,249 elderly individuals, CVAI exhibited superior predictive ability for diabetes compared to traditional anthropometric measures like BMI and WC. CVAI showed high discriminatory power across sex and age groups, particularly among elderly women [33].

This study is the first to demonstrate the incremental predictive value of CVAI in patients with coexisting T2DM, HF, and CKD using NRI and IDI analyses. CVAI significantly improved risk reclassification beyond LVEF and NT-proBNP, reinforcing the central role of visceral adiposity in the pathophysiology of multimorbidity. Although the wide confidence interval for IDI may reflect sample size limitations, the overall trend supports the clinical utility of CVAI. These quantitative metrics provide empirical evidence for the application of CVAI in risk stratification within this complex patient population. Our subgroup analysis confirmed that CVAI significantly correlates with all-cause mortality risk across sex, age, and NYHA functional class subgroups. The most critical subgroup finding is that CVAI’s predictive value for mortality was significant only in patients with LVEF < 40%, while no significant association was observed in those with LVEF ≥ 40%, with a statistically significant interaction effect (P for interaction = 0.049). This discrepancy likely stems from distinct pathophysiological mechanisms between LVEF subgroups. The phenotype-specific association of CVAI—significant in HFrEF but not in HFpEF—likely reflects the distinct mechanistic pathways linking visceral adiposity to each HF subtype. In HFrEF, the pathophysiology is centered on neurohormonal activation and hemodynamic stress [34]. VAT dysfunction exacerbates this core pathology through lipotoxicity (e.g., ectopic cardiac fat deposition impairing contractility) and an adverse adipokine profile (e.g., increased leptin stimulating sympathetic tone), which CVAI effectively captures [35, 36]. In contrast, HFpEF is primarily driven by a systemic pro-inflammatory state leading to microvascular dysfunction and fibrosis [37, 38]. While VAT is a key source of these inflammatory cytokines (e.g., IL-6, TNF-α), its role is so pervasive in HFpEF that a “ceiling effect” may occur; the metabolic-inflammatory burden is universally high, diminishing CVAI’s power for further risk stratification within this cohort [39]. Thus, CVAI serves as a strong marker of a treatable metabolic trait in HFrEF, whereas risk assessment in HFpEF may require biomarkers targeting downstream inflammatory or fibrotic consequences. Consequently, strict CVAI monitoring is warranted in HFrEF patients, enabling early intervention for those with high values. In contrast, HFpEF may require more complex metabolomic assessments.

## Limitation

This study has several limitations that should be considered:

The single-center, retrospective design introduces potential selection bias and unmeasured confounding, despite comprehensive statistical adjustment.

Data on key cardiorenal-protective medications (e.g., SGLT2 inhibitors, GLP-1 receptor agonists) were not available, which may influence the observed associations.

CVAI was calculated from a single baseline measurement; the lack of serial data precludes assessment of how dynamic changes in visceral adiposity influence risk.

CVAI is a surrogate marker for visceral adipose tissue, which was not directly quantified via gold-standard imaging techniques like computed tomography or magnetic resonance imaging.

The sample size, particularly for the LVEF ≥ 40% subgroup, was limited, potentially reducing the power to detect significant associations in this phenotype.

Generalizability may be limited as the study was conducted in a single region of China, and CVAI, developed for Chinese populations, may require validation in other ethnic groups.

## Conclusion

In T2DM-HF-CKD patients, CVAI independently predicted all-cause mortality after comprehensive adjustment for risk factors. Its prognostic value was heart failure phenotype-dependent: significant in HFrEF (LVEF < 40%) but absent in preserved ejection fraction. This highlights distinct pathological contributions of visceral adiposity across HF subtypes. Finally, it is crucial to emphasize that our study design can only establish CVAI as an association risk marker for mortality, not a causal modifiable target. Whether intentional reduction of visceral adiposity, as reflected by a decrease in CVAI, would subsequently translate into improved survival outcomes in this high-risk population requires further investigation through prospective interventional trials.

## Supplementary Information

Supplementary Material 1

## Data Availability

Data cannot be shared publicly due to restrictions under China’s Personal Information Protection Law. De-identified data are available from the corresponding authors (Zhenyan Fu or Yitong Ma) upon reasonable request and with approval from the ethics committee.
