# Supplementary material for: Chinese visceral adiposity index predicts all-cause mortality in patients with type 2 diabetes, heart failure, and chronic kidney disease: a retrospective cohort study
Source: BMC Res Notes. 2025 Oct 29;18:458. doi: 10.1186/s13104-025-07531-6 (PMC12574291; doi:10.1186/s13104-025-07531-6)
Supplement: Supplementary file 1 — Supplementary Material 1 [file 13104_2025_7531_MOESM1_ESM.docx]

Supplementary Table 1. Association of CVAI Tertiles with All-Cause and Cardiovascular Mortality: Cox Proportional Hazards vs. Fine-Gray Competing Risk Models

|  | HR (95%CI) | P | sHR (95%CI) | P |
| --- | --- | --- | --- | --- |
| Tertile 1 | Ref. |  | Ref. |  |
| Tertile 2 | 1.611 (1.050 - 2.472) | 0.029 | 1.620 (0.987 - 2.650) | 0.056 |
| Tertile 3 | 2.806 (1.848 - 4.261) | <0.001 | 2.806 (1.848 - 4.261) | <0.001 |
| P for trend | <0.001 |  | <0.001 |  |

Abbreviations: CI, confidence interval; HR, hazard ratio; sHR, subdistribution hazard ratio; MI, myocardial infarction; eGFR, estimated glomerular filtration rate; LDL-C, low-density lipoprotein cholesterol; LVEF, left ventricular ejection fraction; NT-proBNP, N-terminal pro-B-type natriuretic peptide; HbA1c, glycated hemoglobin; NYHA, New York Heart Association. The Fine-Gray model evaluates the association with cardiovascular-specific mortality, treating non-cardiovascular death as a competing event.

Supplementary Table 2. Incremental Predictive Value of CVAI Beyond Traditional Risk Factors (NRI and IDI Analysis)

|  | AUC (95%CI) | NRI | 95%CI | IDI | 95%CI |
| --- | --- | --- | --- | --- | --- |
| LVEF | 0.555 (0.502-0.609) | Ref. | | Ref. | |
| LVEF+NT-proBNP | 0.600 (0.547-0.654) | 0.154 | 0.068-0.259 | 0.027 | -0.067-0.083 |
| CVAI+LVEF+NT-proBNP | 0.700 (0.651-0.749) | 0.321 | 0.062-0.413 | 0.123 | 0.013-0.259 |

Abbreviations: AUC, area under the curve; CI, confidence interval; CVAI, Chinese Visceral Adiposity Index; IDI, integrated discrimination improvement; LVEF, left ventricular ejection fraction; NRI, net reclassification improvement; NT-proBNP, N-terminal pro-B-type natriuretic peptide.
